# Effect of support on the performance of PtRu-based catalysts in oxidative steam reforming of ethanol to produce hydrogen

**DOI:** 10.3389/fchem.2022.1079214

**Published:** 2022-12-19

**Authors:** Chih-Wei Tang, Chiu-Hung Liu, Shen-Wei Yu, Chen-Bin Wang

**Affiliations:** ^1^ Department of General Education, Army Academy ROC, Taoyuan, Taiwan; ^2^ Graduate School of National Defense Science, Chung Cheng Institute of Technology, National Defense University, Taoyuan, Taiwan; ^3^ Department of Chemical and Materials Engineering, Chung Cheng Institute of Technology, National Defense University, Taoyuan, Taiwan; ^4^ Undergraduate Degree Program of System Engineering and Technology, National Yang Ming Chiao Tung University, Hsinchu, Taiwan

**Keywords:** oxidative steam reforming of ethanol, H2 production, ZrO2, CeO2, PtRu-based based catalysts, support effect

## Abstract

Oxidative steam reforming of ethanol (OSRE) to produce hydrogen has been investigated over a series of supported PtRu catalysts, with different supports. Bimetallic PtRu-based catalysts were prepared by the impregnation method using H_2_PtCl_6_ and RuCl_3_ as precursors. Six supports (reducible oxides of ZrO_2_, CeO_2_, and Co_3_O_4_, and irreducible oxides of ZnO, Al_2_O_3_, and NiO) were chosen to fabricate bimetallic catalysts. The catalytic performance of the OSRE reaction in the series of PtRu-based samples was evaluated using a fixed-bed flow reactor under atmospheric pressure. In front reaction, the catalyst was pre-activated by reduction under 200°C for 3 h. The gas hourly space velocity was adjusted at 66,000 h^−1^, and the optimal molar ratios of the H_2_O/EtOH and O_2_/EtOH feeds were 4.9 and 0.44, respectively. The results indicated that the PtRu supported on the ZrO_2_ and CeO_2_ exhibited superior catalytic performance in the OSRE reaction under a low temperature (a T_R_ of approximately 320°C) for producing the main products of H_2_ and CO_2_ with lower CO and CH_4_ by-products. Also, it was quite stable during a long time evaluation; the maximum Y_H2_ maintained at 4.5–4.2, and the CO distribution approached 3.3–3.5 mol% around 84 h test at 340°C over the PtRu/ZrO_2_ catalyst.

## 1 Introduction

Hydrogen is a versatile element that is often used as a raw material for gasoline (the processes of hydrotreating and hydrocracking), chemistry (the synthesis of NH_3_ and CH_3_OH), foodstuff processing (hydrogenation of fats and oils), steel, and the manufacturing of electronics (Ramachandran and Menon, 1998; Armor, 1999). However, current industry does not produce hydrogen as an energy carrier or as a fuel for generators. In order to support sustainable global economic growth as well as reduce air pollution and the greenhouse effect, it is urgent to adopt hydrogen as an energy carrier; however, several technical hurdles must be conquered to manufacture and transfer the amount of hydrogen needed to reach a hydrogen-based economy.

Hydrogen can be manufactured from oxygenates, such as various ethanol reforming processes (Goldemberg, 2007; Ni et al., 2007; de la Piscina and Homs, 2008; Rabenstein and Hacker, 2008; Zanchet et al., 2015; Li et al., 2016) including the partial oxidation of ethanol (POE), steam reforming of ethanol (SRE), and oxidative steam reforming of ethanol (OSRE). The OSRE reaction is the incorporation of partial oxidation and steam reforming processes. Moreover, the OSRE reaction can lessen the energy of the SRE process and lower the rate of coke formation. Thermodynamic analysis of the production of hydrogen from ethanol by catalytic processes has found that the OSRE process possesses many advantages in terms of heat management and reforming efficiency (Rabenstein and Hacker, 2008).

Besides choosing a single active component (a noble or non-noble metal) as the reforming catalyst, multi-component catalysts have been reported in various catalytic reactions (Bi et al., 2007a; Alayoglu et al., 2008; Pereira et al., 2008; Koh et al., 2009; Pereira et al., 2010). Pereira et al. (Pereira et al., 2010) stated that the K-promoter enhances the catalytic behavior of CoRh/CeZr under the OSRE process to produce hydrogen, in which the addition of Rh facilitates the reducibility of Co. and enhances the catalytic activity. The same group also reported the effect of adding Ru and Rh to the Co./SiO_2_ catalysts and found a synergistic effect between Co and Ru (or Rh) in the OSRE reaction (Pereira et al., 2008). In particular, bimetallic PtRu catalysts have been widely used as CO tolerant for polymer electrolyte fuel cells (PEFCs) (Alayoglu et al., 2008). An investigation on the catalytic ability of PtRu-based catalysts for hydrogen production in reforming reactions confirmed that PtRu/ZrO_2_ is an excellent catalyst for low temperature OSRE reaction (Bi et al., 2007a). A nano-sized PtRu/Al_2_O_3_ originated from the organometallic clusters, which exhibited a high capability to manufacture hydrogen under the SRE reaction (Koh et al., 2009).

The performance of OSRE not only depends on the active metals; the other key factor is the nature of the support. To enhance the catalytic behavior of supported metal catalysts, support has influenced the catalytic performance (Llorca et al., 2002; Furtado et al., 2009; Youn et al., 2010; Wang and Spivey, 2015; Zhang et al., 2015; Tóth et al., 2016; Hsia et al., 2019). The effect of the support’s (ZnO, MgO, ZrO_2_, TiO_2_, and Al_2_O_3_) acidity on the OSRE activity of supported nickel catalysts was explored by Youn et al. (Youn et al., 2010), in which the intermediate acidity of Ni/Ti_0.2_Zr_0.8_O_2_ was identified as the optimal catalyst. The evaluation of the support materials (Al_2_O_3_, Nb_2_O_5_, and Ce_0.6_Zr_0.4_O_2_) of a bimetallic NiCu-based catalyst on the SRE reaction showed that the NiCu/Ce_0.6_Zr_0.4_O_2_ could achieve the best performance for hydrogen production (Furtado et al., 2009).

In our previous work, a PtRu/ZrO_2_ catalyst modified with Na in an OSRE indicates active at 300°C and begets little CO at 340°C (Wang et al., 2011). Here in, the OSRE reaction was further studied over series PtRu-based catalysts. Six supports (reducible oxides of ZrO_2_, CeO_2_, and Co_3_O_4_, and irreducible oxides of ZnO, Al_2_O_3_, and NiO) were picked to fabricate supported bimetallic catalysts. The goal of this effort was to find an excellent and durable catalyst that could be applied to the on-board reforming of ethanol at low temperatures.

## 2 Experimental

### 2.1 Preparation of catalysts

Six supports (ZrO_2_, CeO_2_, Co_3_O_4_, ZnO, Al_2_O_3_, and NiO) were chosen to prepare the supported bimetallic catalysts. The Al_2_O_3_ was purchased from Merck (γ-form, 142 m^2^⋅g^−1^), while the others were self-synthesized. The ZrO_2_ (130 m^2^⋅g^−1^) was prepared using the sol-gel method (Bi et al., 2007a), the CeO_2_ (106 m^2^⋅g^−1^) was prepared using a simple reduction-oxidation method (Siang et al., 2010), the Co_3_O_4_ (102 m^2^⋅g^−1^) and NiO (104 m^2^⋅g^−1^) were prepared by the precipitation-oxidation method (Wang et al., 2005a; Lai et al., 2006), and the ZnO (89 m^2^⋅g^−1^) was obtained through the thermal decomposition of Zn(OH)_2_·2ZnCO_3_·xH_2_O (Stream) in the air (Chiou et al., 2012), respectively. The supported PtRu catalysts were prepared by the impregnation method using an aqueous solution of H_2_PtCl_6_ (Merck) and RuCl_3_ (Strem) as precursors (the loading of each component was 1.5 wt%). The as-prepared sample went through drying at 110°C and calcination at 400°C for 4 h in the air, after which the fabricated samples were smashed to 60–80 mesh and kept as fresh catalysts (both metal content and surface area are listed in Table 1).

**TABLE 1:** Surface area and metal content of series PtRu-based catalysts.

Catalyst	S_BET_ (m^2^⋅g^−1^)	Metal content (wt%)	
		Pt	Ru
PtRu/ZrO_2_	116	1.36	1.28
PtRu/CeO_2_	104	1.38	1.34
PtRu/Co_3_O_4_	86	1.31	1.26
PtRu/Al_2_O_3_	131	1.41	1.38
PtRu/ZnO	58	1.34	1.33
PtRu/NiO	92	1.39	1.42

### 2.2 Physicochemical characterizations

Both the Pt and the Ru contents in the fresh catalysts were ascertained by the measurement of ICP-MS (Perkin-Elmer). The surface area (S_BET_) was measured with the physisorption using a Micromeritics ASAP 2010 instrument. First, the catalysts were pre-outgassed in a vacuum for 3 h at 110°C, then, the S_BET_ was measured by the N_2_ adsorption at 77 K. The phases and crystalline structures were surveyed with X-ray diffraction (XRD) using a MAC Science MXP18 diffractometer with Cu K_α1_ radiation (*λ* = 1.5405 Ǻ) at 30 mA and 40 kV. The particle size was observed using transmission electron microscopy (TEM) images from a JEM-2010 transmission electron microscope (JEOL) with a 200 kV acceleration voltage. During the TPR experiment, a 50 mg sample located in the reactor and was heated at a ramping rate of 7°C⋅min^−1^ from RT to 500°C under a reducing gas (10% H_2_/N_2_) with a flow rate of 10 ml⋅min^−1^. The consumption of H_2_ was detected continuously using a thermal conductivity detector (TCD).

### 2.3 Activity test of catalysts

A fixed-bed flow reactor was chosen to evaluate the performance of the PtRu-based catalysts towards the OSRE reaction under atmospheric pressure. A 100-mg sample was placed in a quartz tubular reactor (4 mm i. d.) and guarded with glass-wool plugs. The reactor was twined with heating tape and the temperature was controlled using a thermocouple (1.2 mm i. d.) placed in the center of the reactor bed. Prior to the reaction, the catalyst was pre-activated by reduction at 200°C for 3 h. The catalytic activity of the series of supported PtRu catalysts towards OSRE was executed by tuning the molar ratio of the H_2_O/EtOH (0.8–13) and O_2_/EtOH (0.32–0.61), which was executed between 280°C and 600°C under atmospheric pressure. The outlet gas was detected by both gas chromatography (GC), one with an MS-5A column (for the separation of H_2_, O_2_, CH_4_, and CO), and the other with a Porapak Q column (for the separation of C_2_H_5_OH, H_2_O, CH_3_CHO, CH_3_COOC_2_H_5_, and CO_2_). Based on the ethanol conversion (X_EtOH_), selectivity of carbon monoxide (S_CO_) and yield of hydrogen (Y_H2_) to evaluate the catalytic performance as follows:

XEtOH%=[nEtOHin– n EtOHout]n EtOHin×100% (1)

SCO%=nCOoutn CH4out+n COout+n CO2out×100% (2)

YH2=nH2outnEtOHin– n EtOHout (3)

## 3 Results and discussion

### 3.1 Characterization of catalysts

**FIGURE 1:**
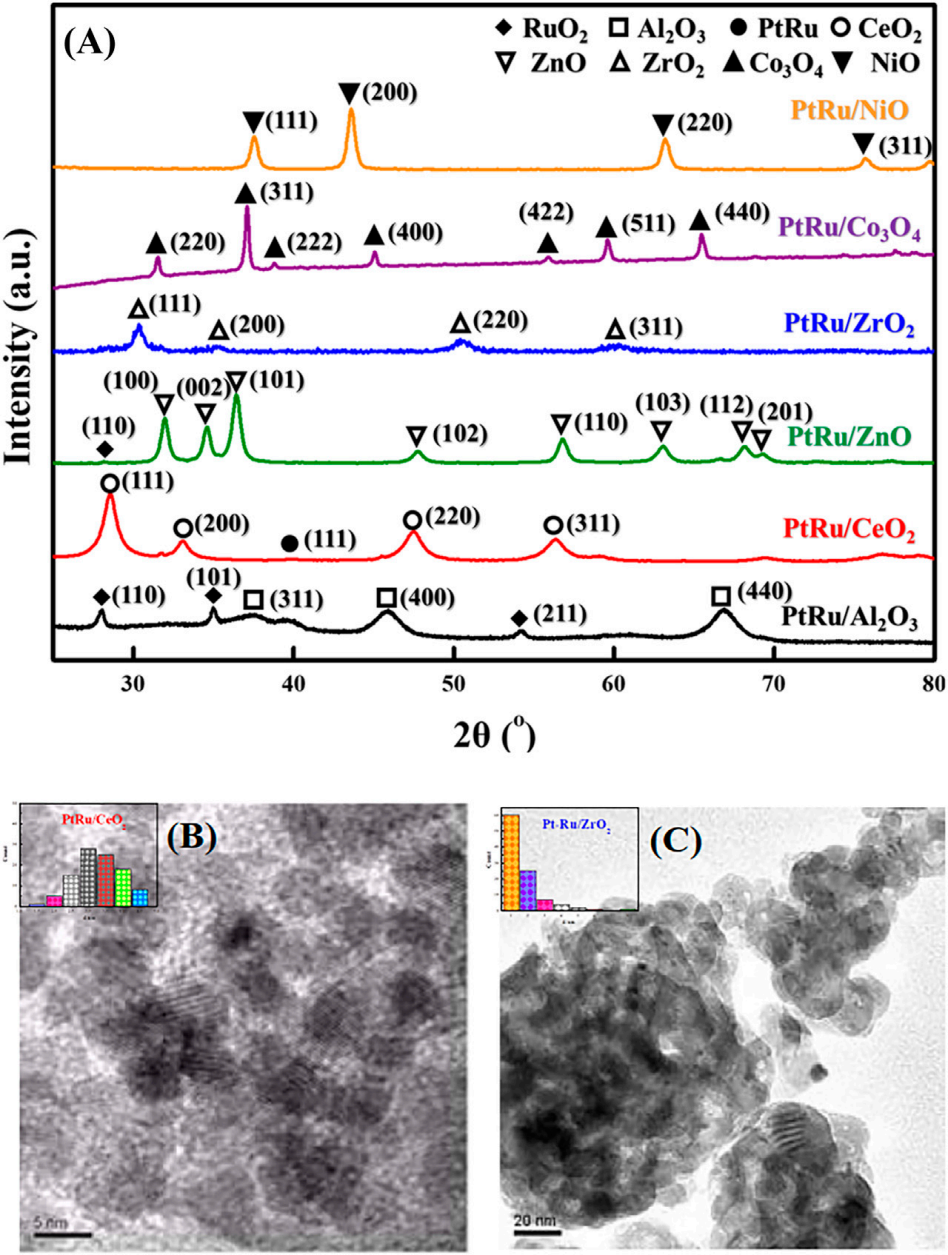
XRD patterns and TEM images of series PtRu-based catalysts: **(A)** XRD profile **(B)** TEM image of PtRu/CeO_2_ catalyst **(C)** TEM image of PtRu/ZrO_2_ catalyst.

**FIGURE 2:**
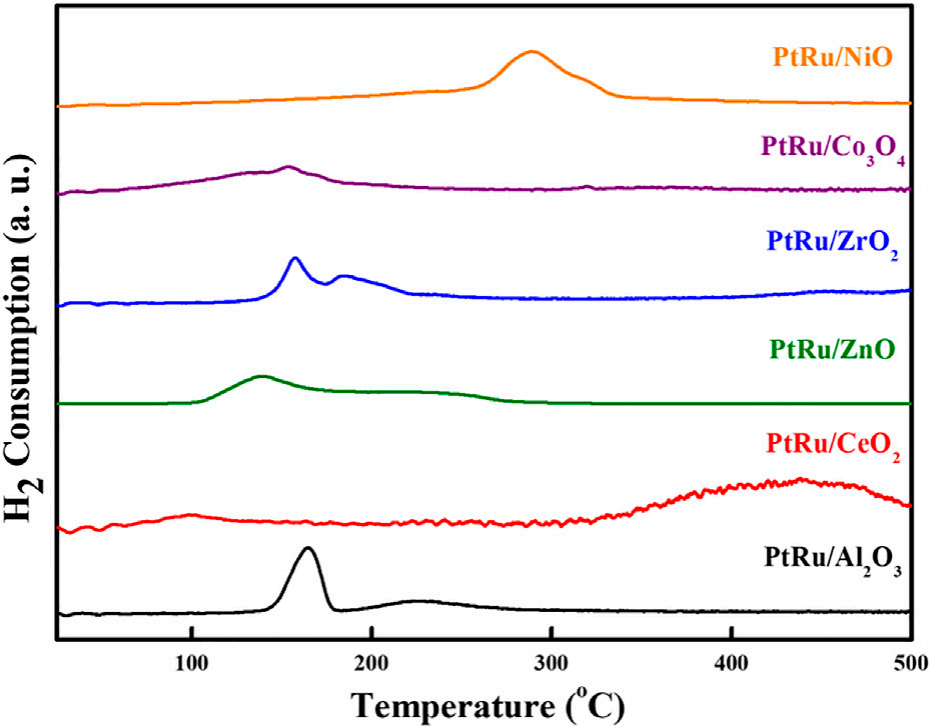
TPR profiles of series PtRu-based catalysts.

Figure 1A indicates the XRD patterns of the six bimetallic PtRu catalysts. Except for the PtRu/Al_2_O_3_ and PtRu/ZnO catalysts, which exhibited a very faint RuO_2_ phase, no significant diffraction peak of PtRu (111) was observed in all catalysts. This result demonstrated that the nanoparticles of the bimetallic PtRu were smaller than 5 nm. The crystal phase of metal oxides is conspicuous, i.e. the unique diffraction peaks of PtRu/NiO at 37.6^o^, 43.6^o^, 63.2^o^, and 75.7^o^, corresponding to the (111), (200), (220) and (311) planes, respectively, were attributed to the cubic NiO (JCPDS 04-0835); the clear diffraction peaks of PtRu/Co_3_O_4_ at 31.5^o^, 37.1^o^, 38.8^o^, 45.1^o^, 55.9^o^, 59.6^o^ and 65.5^o^, corresponding to the (220), (311), (222), (400), (422), (511) and (440) planes, respectively, were ascribed to the spinel Co_3_O_4_ (JCPDS 43-1003); the noticeable diffraction peaks of PtRu/ZrO_2_ at 30.4^o^, 35.4^o^, 50.4^o^ and 60.4^o^, corresponding to the (111), (200), (220) and (311) planes, respectively, were associated with the tetragonal ZrO_2_ (JCPDS 79-1769); the distinct diffraction peaks of PtRu/ZnO at 31.9^o^, 34.6^o^, 36.4^o^, 47.8^o^, 56.8^o^, 63.1^o^, 68.2^o^ and 69.3^o^, corresponding to the (100), (002), (101), (102), (110), (103), (112) and (201) planes respectively, were well indexed to the hexagonal ZnO (JCPDS 79-2205); the bright diffraction peaks of PtRu/CeO_2_ at 28.5^o^, 33.1^o^, 47.5^o^ and 56.4^o^ corresponding to the (111), (200), (220) and (311) planes, respectively, were confirmed the cubic CeO_2_ (JCPDS 34-0394); the bright diffraction peaks of PtRu/Al_2_O_3_ at 37.3^o^, 45.9^o^ and 66.9^o^, corresponding to the (311), (400) and (440) planes, respectively, were assigned to the γ-Al_2_O_3_ (JCPDS 10-0425). Figures 1B, C present the TEM images of the PtRu/CeO_2_ and PtRu/ZrO_2_, in which the average particle sizes of the PtRu were around 3.3 and 1.7 nm for both catalysts, respectively. The reducibility of the bimetallic PtRu catalysts was evaluated using temperature programmed reduction experiments (the TPR profile is listed in Figure 2). According to the literature (Trovarelli, 1996; Lin et al., 2003; Wang et al., 2005a; Wang et al., 2005b; Lai et al., 2006; Siang et al., 2010), the reduction of NiO occurs around 300°C (Wang et al., 2005b; Lai et al., 2006), the reduction of Co_3_O_4_ occurs around 200–400°C (Lin et al., 2003; Wang et al., 2005a), and the reduction of the surface capping oxygen ions of CeO_2_ occurs above 400°C (Trovarelli, 1996; Siang et al., 2010). The results showed that in addition to the reduction of the active species (the bimetallic oxide reduced at below 200°C for all catalysts and the RuO_2_ reduced at around 200°C–300°C for the PtRu/Al_2_O_3_ and PtRu/ZnO catalysts) (Bi et al., 2007a), the NiO, Co_3_O_4_, and the surface oxygen of the CeO_2_ supports could also be reduced at temperatures below 500°C.

### 3.2 Catalytic activity of oxidative steam reforming of ethanol reaction

#### 3.2.1 Effect of feed molar ratio

For the purpose of verification, the optimal feeds on the molar ratio of H_2_O/EtOH and O_2_/EtOH, the PtRu/ZrO_2_ sample has been chosen to tune both H_2_O/EtOH and O_2_/EtOH molar ratio, separately. First, a regular H_2_O/EtOH molar ratio of 4.9 was transmitted to the reactor to adjust the O_2_/EtOH molar ratio (0.32, 0.44, and 0.61, respectively). All situations revealed a complete conversion at and above 300°C. The distribution of products and the hydrogen yield are displayed in Table 2, which shows that the diminishing of the O_2_/EtOH ratio inclines to grow in number the amount of by-products (CO and CH_4_) and lessened the hydrogen yield from 280°C to 320°C. However, the raising of the O_2_/EtOH molar ratio inclines to facilitate the oxidation of hydrogen and reduced the hydrogen yield. Based on the tuning of O_2_/EtOH, 0.44 was the most appropriate molar ratio. Under this circumstance, both the Y_H2_ improved and the CO reduced with the raising of the reaction temperature (T_R_). Second, a definite O_2_/EtOH molar ratio of 0.44 was delivered to the reactor to regulate the H_2_O/EtOH molar ratio (0.8, 2.2, 4.9, and 13, respectively). The product distribution and hydrogen yield are exhibited in Table 3, which reveals that the diminishing of the H_2_O/EtOH ratio inclines to boost the POE reaction, while the raising of the H_2_O/EtOH ratio stimulates produce more CO side-product. According to the regulating of the H_2_O/EtOH, 4.9 was the most appropriate molar ratio for the OSRE reaction, as it enhanced the hydrogen yield and reduced the CO distribution manifestly from 280°C to 320°C.

**TABLE 2:** Effect of O_2_/EtOH for OSRE reaction with various temperatures over the PtRu/ZrO_2_ catalyst under a molar ratio of H_2_O/EtOH = 4.9.

O_2_/EtOH	T_R_ (°C)	Ethanol conversion (X_EtOH_, %)/Products distribution (mol%)					Y_H2_
		X_EtOH_	H_2_	CH_4_	CO	CO_2_	
0.32	280	100	47.6	12.1	16.7	23.6	1.8
	300	100	47.1	13.3	16.1	23.5	1.8
	320	100	46.7	15.4	15.5	22.4	1.8
0.44	280	100	64.1	2.3	11.0	22.6	3.3
	300	100	64.9	3.4	8.6	23.1	3.4
	320	100	71.4	1.2	3.4	24.0	4.2
0.61	280	100	60.3	6.9	12.1	20.7	2.1
	300	100	63.0	4.2	9.5	23.3	2.8
	320	100	63.4	2.4	10.6	23.6	2.9

**TABLE 3:** Effect of H_2_O/EtOH for OSRE reaction with various temperatures over the PtRu/ZrO_2_ catalyst under a molar ratio of O_2_/EtOH = 0.44.

H_2_O/EtOH	T_R_ (°C)	Ethanol conversion (X_EtOH_, %)/Products distribution (mol%)					Y_H2_
		X_EtOH_	H_2_	CH_4_	CO	CO_2_	
13	280	100	66.7	0	10.8	22.5	2.5
	300	100	66.8	0	10.7	22.5	2.9
	320	100	66.7	0	10.1	23.2	3.0
4.9	280	100	64.1	2.3	11.0	22.6	3.3
	300	100	64.9	3.4	8.6	23.1	3.4
	320	100	71.4	1.2	3.4	24.0	4.2
2.2	350	100	57.9	14.8	8.1	19.2	2.1
	370	100	60.1	16.0	7.8	16.1	2.2
	390	100	60.3	16.7	3.9	19.1	2.4
0.8	350	100	60.5	13.9	3.7	21.9	2.6
	370	100	61.2	18.5	3.5	16.8	2.2
	390	100	61.6	20.1	5.5	12.8	1.9

#### 3.2.2 Effect of support

**FIGURE 3:**
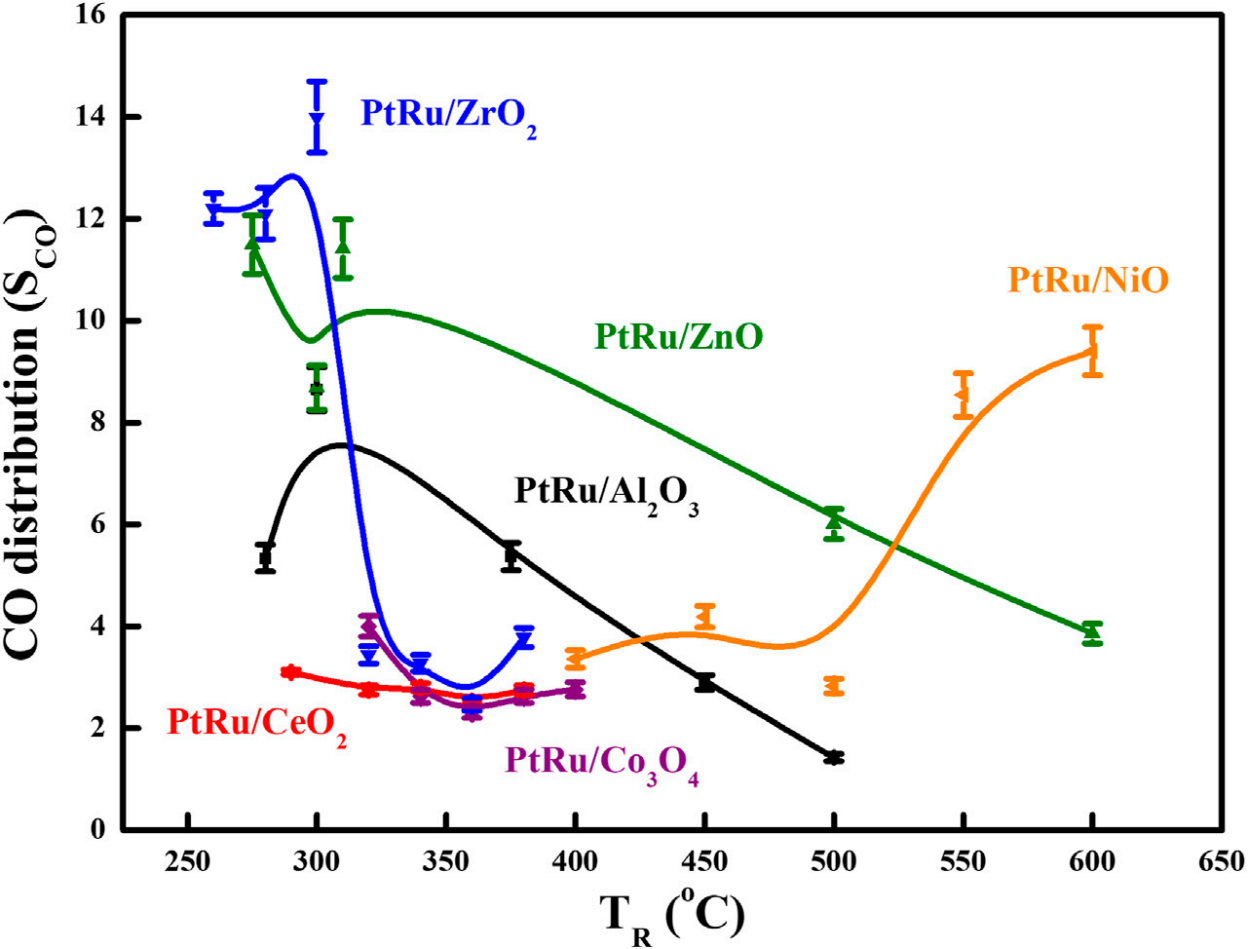
S_CO_ of series PtRu-based catalysts for OSRE reaction under various temperatures.

**FIGURE 4:**
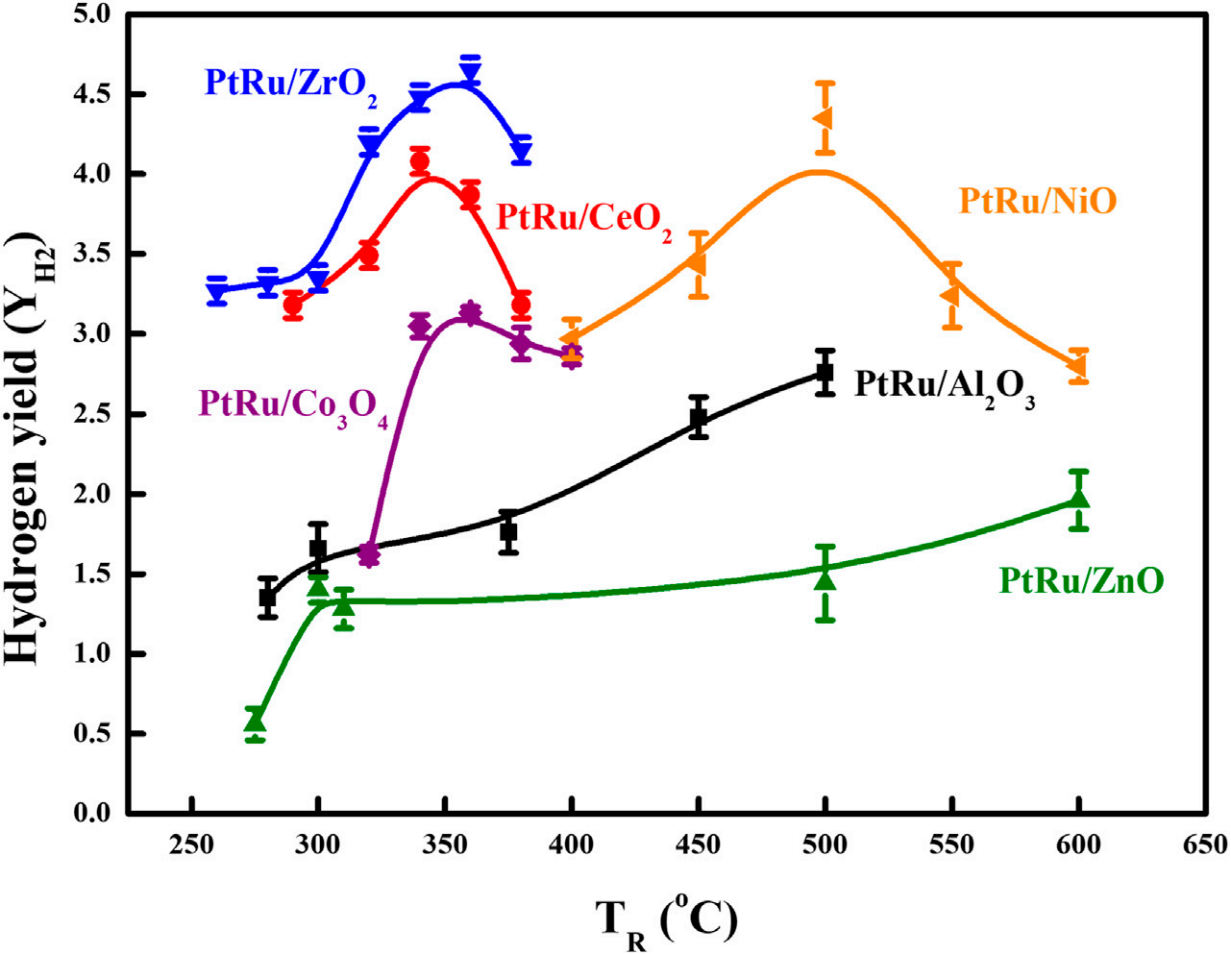
Y_H2_ of series PtRu-based catalysts for OSRE reaction under various temperatures.

The literature (Mavrikakis and Barteau, 1998; Cavallaro et al., 2003; Fierro et al., 2005; Diagne et al., 2002) has reported that maintaining the OSRE process at a low temperature can preclude methanation (at an adequate temperature of around 500°C) and initiate large amounts of CO (at an adequate temperature of around 700°C). Based on the above consequents, the optimized feeds of the O_2_/EtOH and H_2_O/EtOH on the OSRE reaction were 0.44 and 4.9, respectively. Keeping this reforming condition, six bimetallic PtRu-based catalysts have been evaluated. Table 4 summarizes the conversion and distribution of products, and Figures 3, 4 gather up the Y_H2_ and S_CO_ over the series of PtRu-based catalysts for the OSRE reaction under various temperatures. The curves in Figures 3, 4 were drawn based on the fitting algorithm of the B-spline curve brought into the experimental data. In order to discuss the uncertainty analysis for numerical data, each sample was evaluated three times separately and the results indicated that the error was within ±1%–5%. Apparently, a synergistic effect emerged between the metal oxide supports and PtRu active species. The ethanol could be converted completely at lower temperatures for the reducible oxides (CeO_2_ and ZrO_2_ ≈ 280°C, Co_3_O_4_ > 300°C) supported catalysts with only C_1_ species (CH_4_, CO, and CO_2_), while the ethanol could be converted completely at higher temperatures for the irreducible oxides (Al_2_O_3_ > 350°C, NiO >400°C, and ZnO >450°C) supported catalysts with C_1_ species and a trace amount of undesirable C_2_H_4_ and CH_3_CHO species. Although the catalytic activity of the PtRu/Co_3_O_4_ catalyst was bright and the S_CO_ was lower, the Y_H2_ was also lower than 3, and the long-time reaction impel a deactivation by the progressive deposited carbon. The Y_H2_ was high for the PtRu/NiO catalyst, while the T_R_ approached a high temperature that initiated the decomposition of ethanol and increased the S_CO_ as the T_R_ moved above 500°C. Both the PtRu/Al_2_O_3_ and the PtRu/ZnO catalysts possessed a lower Y_H2_ and higher S_CO_. Bi et al. (Bi et al., 2007b) suggested that the reducible oxides could offer the lattice oxygen (O_L_) to assist the oxidation of adsorbed CO (CO_ad_), and the irreducible oxides lacked the lattice oxygen which made the oxygen molecules had to be adsorbed on the metal surface (O_ad_). Since the O_L_ is more effective than O_ad_ for oxidation of CO_ad_, therefore, a high S_CO_ produced easily from the desorption of CO_ad_ over the irreducible oxides, and the O_ad_ could integrate the adjacent adsorbed hydrogen to lower the Y_H2_. Additionally, both the CeO_2_ and the ZrO_2_ can improve the dispersion of the active phase (Srinivas et al., 2003; Biswas and Kunzru, 2008), and possess large amount of surface oxygen vacancies that can restore/release oxygen (Hori et al., 1998; Silva et al., 2005) to prevent deactivation by the deposited coke and were active in the water gas shift (WGS) reaction (Gutierrez et al., 2011) to elevate the Y_H2_ and lower the S_CO_. Both the Co_3_O_4_ and the NiO have been reported to exhibit the performance C−C bond cleavage on reforming of ethanol and a high Y_H2_, however, both deactivated easily *via* the sintering and/or carbon deposition over the catalyst surface (Abdelkader et al., 2013; Sekine et al., 2009; da Silva et al., 2011). The Al_2_O_3_ has low mobile oxygen vacancies (Duprez, 1997), there is a limited number of oxygen intermediates available for CO oxidation, and the acidic support can stimulate the dehydration of ethanol to ethylene (Fatsikostas and Verykios, 2004), which pursued a high S_CO_ and low Y_H2_ production. Based on the demonstration and comparison of these reported literatures, in this study, both the PtRu/ZrO_2_ and the PtRu/CeO_2_ presented well-dispersed and excellent OSRE catalysts to produce hydrogen under low temperatures. The maximum Y_H2_ approached 4.5 and the S_CO_ was 3.3 mol% at 340°C for the PtRu/ZrO_2_ catalyst, while the Y_H2_ was 4.0 and the CO distribution was 2.8 mol% for the PtRu/CeO_2_ catalyst, respectively.

**TABLE 4:** Catalytic performance in the OSRE reaction over series PtRu-based catalysts under O_2_/EtOH = 0.44 and H_2_O/EtOH = 4.9.

Catalyst	TR(C)	Ethanol conversion (X_EtoH_, %)/Products distribution (no! %)							Y_H2_
		X_E011_	H_2_	CH_4_	CO	CO_2_	CH_2_CH_2_	CH_3_CHO	
PtRu/ZrO_2_	280	100	64.1	2.3	11.0	22.6			3.3
	300	100	64.9	3.4	8.6	23.1			3.4
	320	100	71.4	1.2	3.4	24.0			4.2
	340	100	71.7	1.1	3.3	23.9			4.5
PtRu/CeO_2_	290	100	66.4	6.6	3.1	23.9			3.2
	320	100	68.4	5.5	2.8	23.3			3.5
	340	100	70.0	4.0	2.8	23.2			4.0
	360	100	68.7	5.0	2.5	23.8			3.8
PtRu/Co_3_O_4_	320	100	63.7	9.5	4.0	22.8			1.6
	340	100	67.4	6.8	2.6	23.2			3.1
	360	100	68.1	5.8	2.3	23.8			3.1
	380	100	69.5	5.1	2.6	23.8			2.9
PtRu/Al_2_O_3_	300	95	58.4	8.3	8.7	22.4	2.2		1.7
	375	96	58.7	11.8	5.4	23.1	1.0		1.8
	450	97	61.3	11.5	2.9	24.0	0.3		2.5
	500	97	65.0	9.2	1.4	24.4	0		2.8
PtRu/ZnO	300	93	53.3	10.6	8.7	23.2		4.2	1.4
	310	95	55.0	10.1	9.4	23.0		4.5	1.3
	500	96	54.2	15.2	6.0	23.7		3.9	1.5
	600	98	57.5	14.6	3.9	23.0		1.0	2.9
PtRu/NiO	400	98	65.3	5.3	3.4	23.0		3.0	3.0
	450	98	65.1	6.4	4.2	22.0		2.3	3.4
	500	98	67.9	4.6	2.8	23.5		1.2	4.4
	600	99	64.3	4.4	9.4	21.9		0	2.9

**FIGURE 5:**
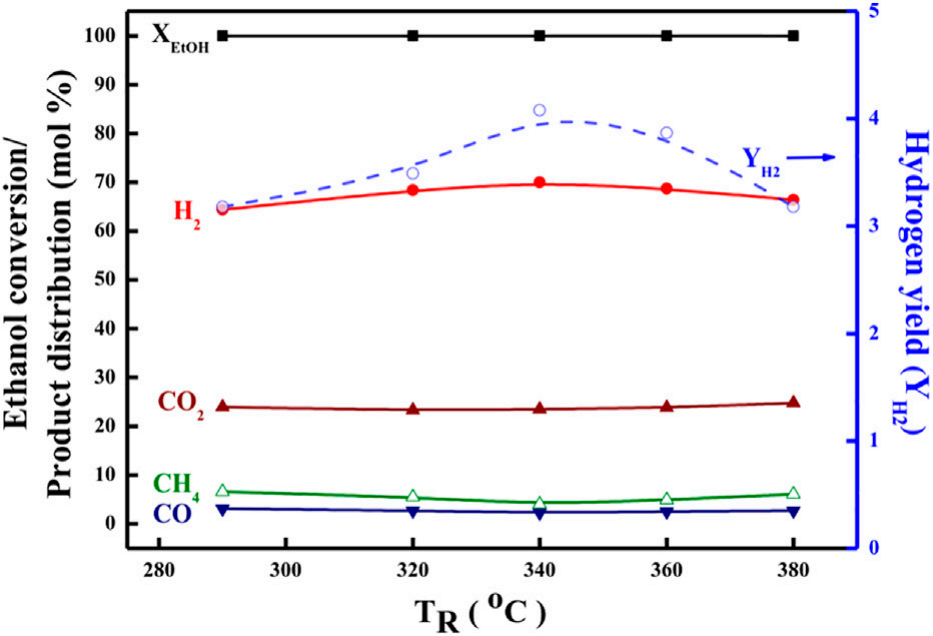
Catalytic performance in the OSRE reaction over the PtRu/CeO_2_ catalyst under O_2_/EtOH = 0.44, H_2_O/EtOH = 4.9.

**FIGURE 6:**
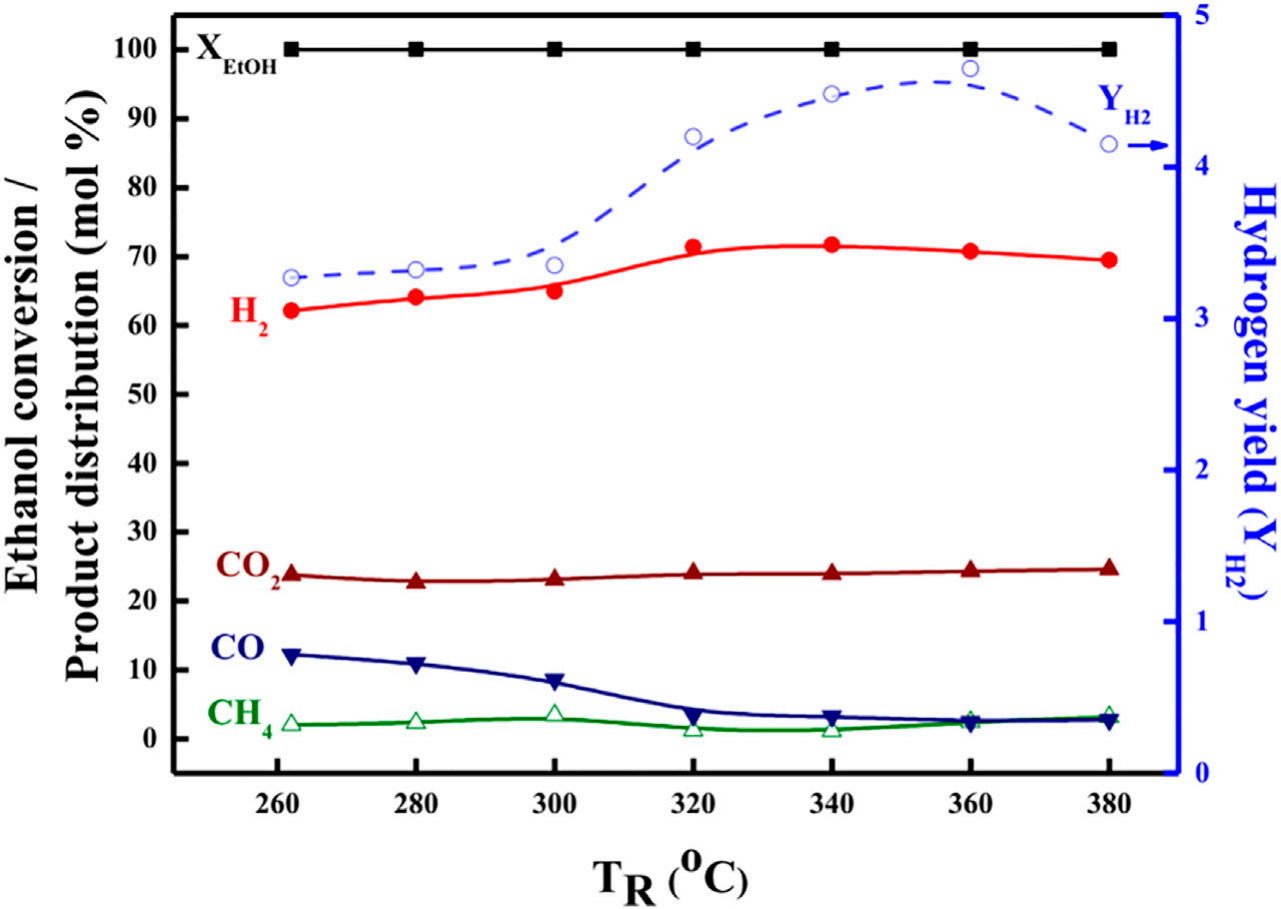
Catalytic performance in the OSRE reaction over the PtRu/ZrO_2_ catalyst under O_2_/EtOH = 0.44, H_2_O/EtOH = 4.9.

**SCHEME 1:**
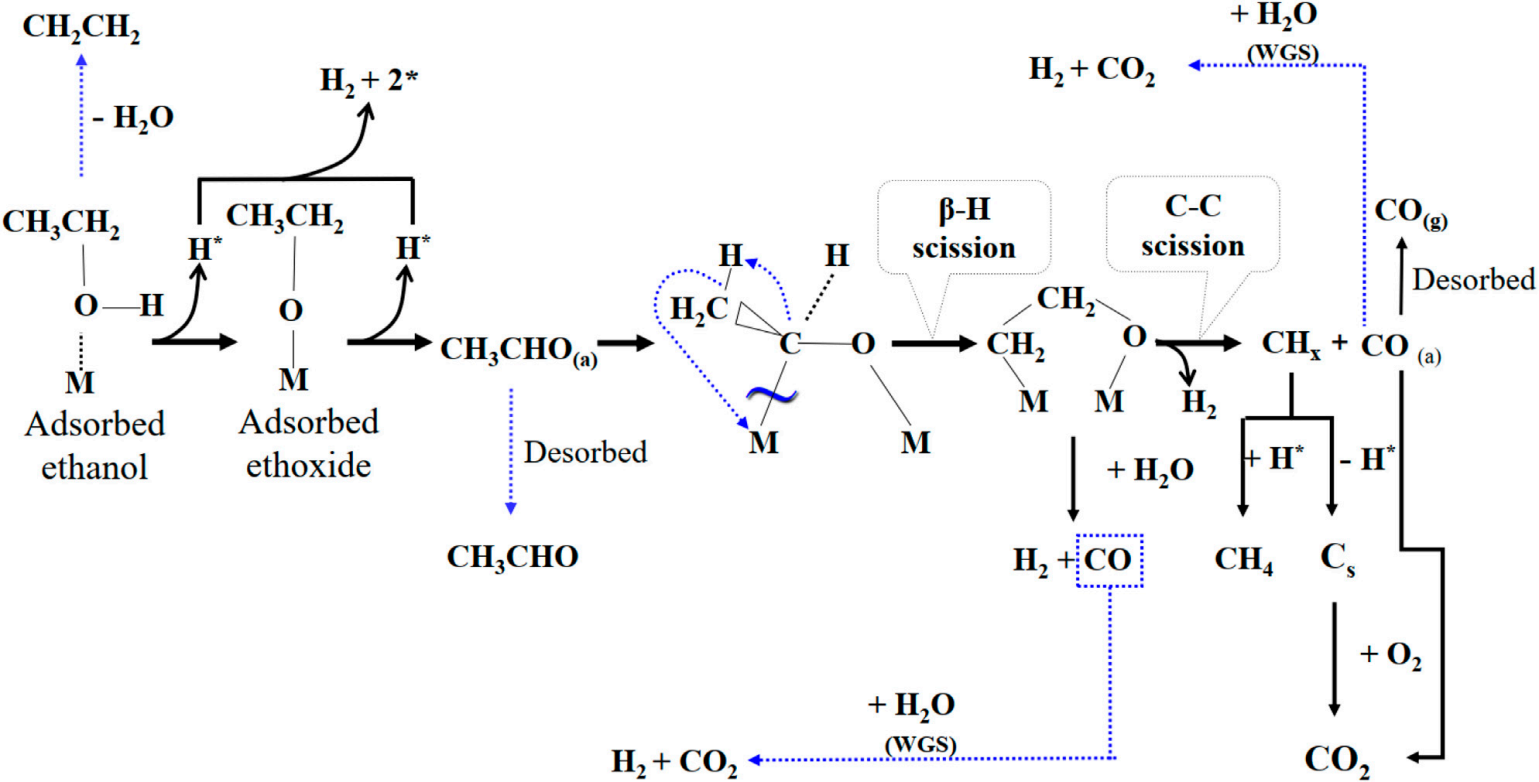
Reaction pathway for the OSRE reaction over the PtRu-based catalysts.

Further, in order to ascertain the features and derivations of the OSRE pathways over PtRu-based catalysts, the ethanol conversion along with the distribution of H_2_, CO_2_, CH_4_, and CO products in the outlet stream as a function of the T_R_ was plotted between 260 and 380°C for both the PtRu/CeO_2_ and the PtRu/ZrO_2_ catalysts, as shown in Figures 5, 6. Summarizing with Table 4, also, comparing with other studies (Rabenstein and Hacker, 2008; Bi et al., 2007a; Mavrikakis and Barteau, 1998; Hung et al., 2012), the simplified reaction network in the OSRE process over the PtRu-based catalysts was outlined in Scheme 1. The involved intermediate steps included a variety of reactions which depended on the dependency relationships of different functionalities of the catalyst. Only minor ethylene produced over the PtRu/Al_2_O_3_ catalyst *via* the dehydration of adsorbed ethanol. All catalysts showed that the ethanol could be converted completely over the whole temperature range, and the consumption of oxygen was complete. The products of the H_2_ and CO_2_ increased with the T_R_. Thus, the performance at relatively low temperatures (<300°C) was mainly due to the partial oxidation of ethanol by consuming the O_2_ according to Eq. 4:

C2H5OH+32O2→2CO2+3H2 (4)

The key step of ethanol reforming is the cracking of the C−C bond. The C−C bond can be cracked easily through the use of ruthenium and platinum (Davda et al., 2005; Zhao et al., 2019; Rajabi et al., 2021; Zare et al., 2021). The initial partial ethanol oxidation provided the potential for the dehydrogenation of the ethanol (Eqs 5, 6) and its decarbonylation (Eq. 8) over the PtRu/ZrO_2_ and PtRu/CeO_2_ catalysts are quicker than PtRu/ZnO and PtRu/NiO (where * is the active site), where the later produced a minor CH_3_CHO *via* desorption of adsorbed acetaldehyde.

$$C_{2}H_{5}OH+*\to {CH}_{3}CHO-*+H_{2}$$ (5)

$${CH}_{3}CHO-*\to {CH}_{3}CO-*+1/2H_{2}$$ (6)

$${CH}_{3}CHO-*\to {CH}_{3}CHO+*$$ (7)

$${CH}_{3}CO-*+*\to CO-*+{CH}_{3}-*$$ (8)

The amount of CH_4_ was lower on both PtRu/ZrO_2_ and PtRu/CeO_2_ catalysts, indicating that the sequent steam reforming of the methyl (Eq. 9) after the splitting of the acetyl group (CH_3_CO) was preferential over the formation of methane (Eq. 10).

$${CH}_{3}-*+H_{2}O\to CO+5/2H_{2}$$ (9)

$${CH}_{3}-*+H-*\to {CH}_{4}+2*$$ (10)

The S_CO_ over PtRu/CeO_2_ was lower than PtRu/ZrO_2_ catalyst, while the CO distribution over PtRu/ZrO_2_ declined abruptly and accompanied the increase of CO_2_ and H_2_ as the T_R_ upon 300°C. Also, the amount of CH_4_ grew and accompanied the decrease of H_2_ as the T_R_ moved above 360°C. In accordance with these observations, the PtRu/CeO_2_ catalyst could preferentially oxidize the CO (Eq. 11) in the hydrogen-rich gases at a lower T_R_. The WGS (Eq. 12) reaction was favorable compared to the PtRu/ZrO_2_ catalyst at temperatures above 300°C for lowering the amount of CO, and the increase of CH_4_ coupled with the decrease of H_2_ upon 360°C was attributed to the methanation of CO or CO_2_ (Eq. 13).

$$CO-*\left(H_{2}\right)+O-*\to {CO}_{2}\left(H_{2}\right)+2*$$ (11)

$$CO-*+H_{2}O\to {CO}_{2}+H_{2}+*$$ (12)

$$CO-*+3H_{2}\to {CH}_{4}+H_{2}O+*$$ (13)

#### 3.2.3 Catalytic stability

**FIGURE 7:**
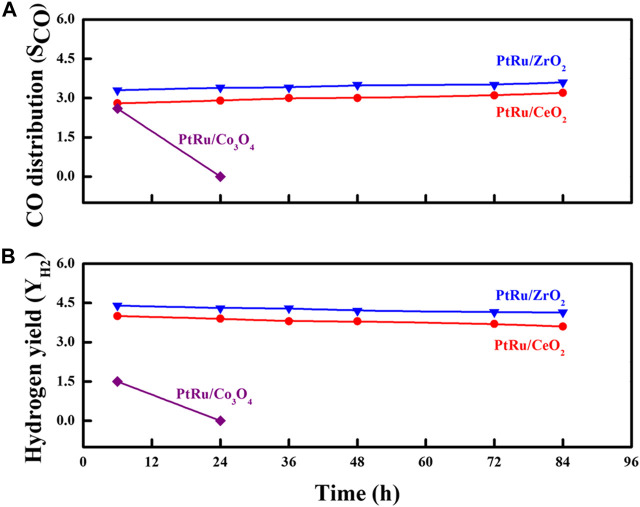
Durability test for the OSRE reaction over PtRu/ZrO_2_, PtRu/CeO_2_ and PtRu/Co_3_O_4_ catalysts: **(A)** S_CO_ **(B)** Y_H2_.

**FIGURE 8:**
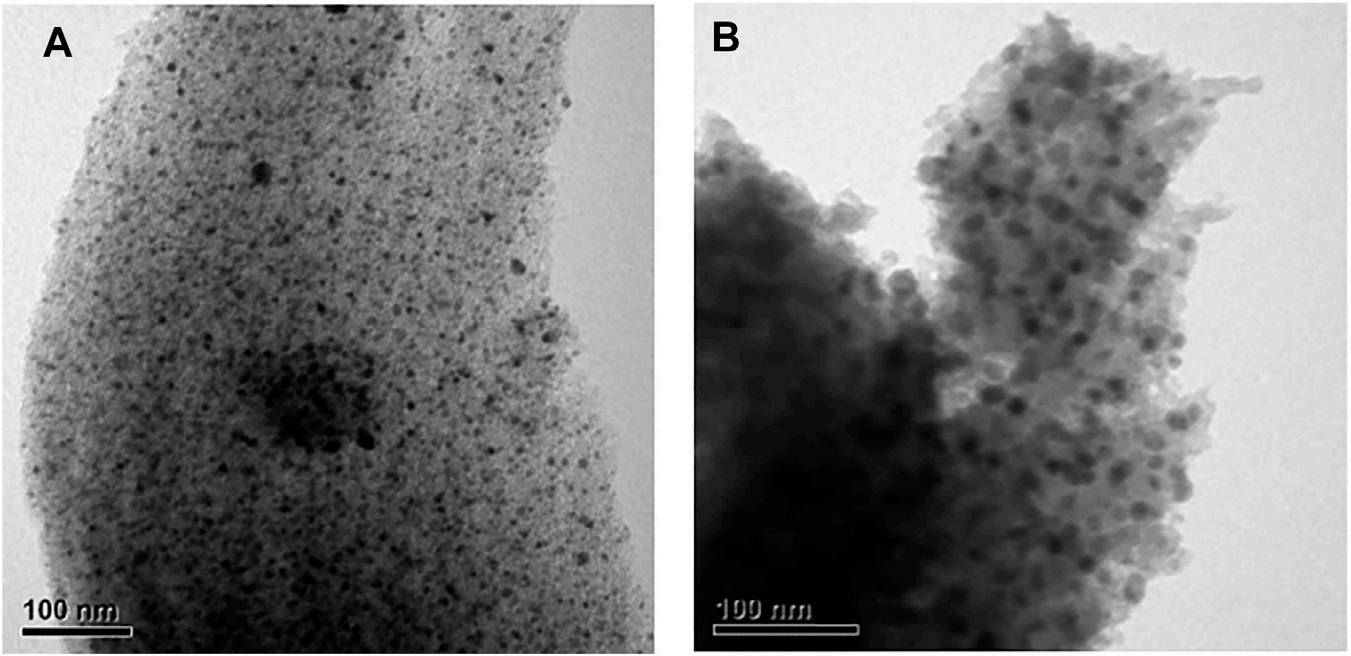
TEM images of PtRu/Co_3_O_4_ catalyst: **(A)** Fresh **(B)** Spent.

**FIGURE 9:**
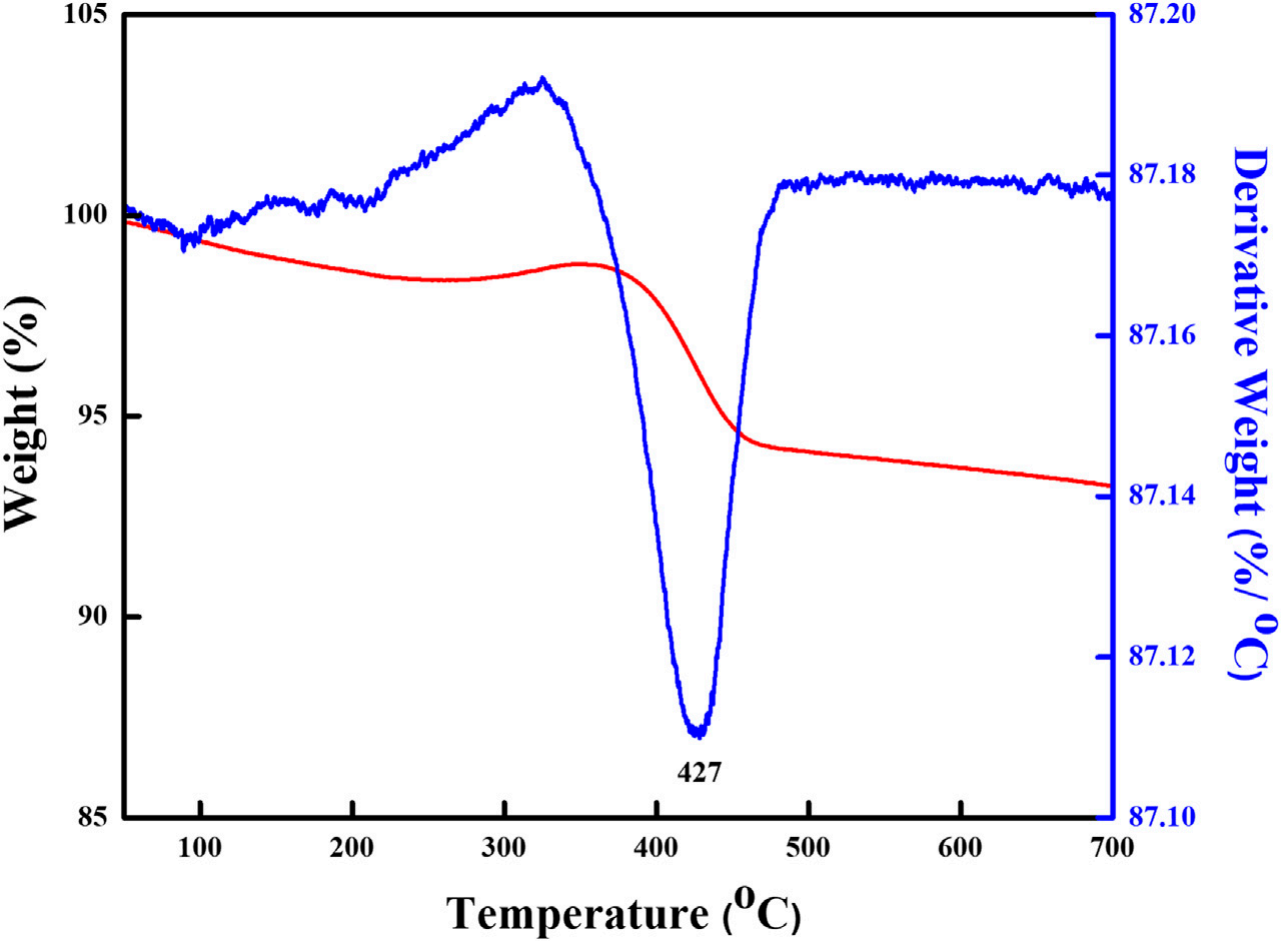
TG/DTG profiles of spent PtRu/Co_3_O_4_ catalyst.

Stability is a judgmental feature of any heterogeneous catalyst that decides its efficiency in a catalytic reaction. In order to highlight the discrepancy in the Y_H2_ and S_CO_ over the three reducible oxide-supported PtRu catalysts for the OSRE reaction, the evaluation of the catalyst stability was carried out as a function of the time-on-flux at 340°C, as shown in Figure 7. Except for the PtRu/Co_3_O_4_ catalyst, in which the activity decayed quickly with the reaction time, the conversion of the EtOH remained complete within the test over PtRu/ZrO_2_ and PtRu/CeO_2_ catalysts. Under the OSRE reaction, the deactivation of the Co-based catalyst could be attributed to the deposited coke, sintering of the active phase, and the encapsulation of metal sites into the support (da Silva et al., 2010; Espitia-Sibaja et al., 2017). Figure 8 displays the TEM image of the fresh and spent PtRu/Co_3_O_4_ catalyst. Apparently, the well-dispersed PtRu nanoparticles were somewhat agglomerated and capsulated by the amorphous carbon through the OSRE reaction over the PtRu/Co_3_O_4_ catalyst. To affirm the amorphous carbon, further analysis with thermal analysis, and the TG/DTG profile of spent PtRu/Co_3_O_4_ catalyst was listed in Figure 9. Apparently, the deposited carbon (∼6.5%wt loss) could be oxidized around 427°C. Both the PtRu/ZrO_2_ and PtRu/CeO_2_ catalysts showed preferential durability and exceeded 80 h of stability, whereas the PtRu/Co_3_O_4_ catalyst was deactivated within 20 h by the deposited carbon. The maximum Y_H2_ was maintained at 4.5–4.2 and the CO distribution approached 3.3–3.5 mol% on the PtRu/ZrO_2_ catalyst. The maximum Y_H2_ stayed at 4.0–3.6 and the CO distribution approached 2.8–3.2 mol% over the PtRu/CeO_2_ catalyst, respectively, around 84 h test at 340. Many reactions are associated with the OSRE, the aims to pursue a high Y_H2_ while generating minimum CO. Greluk et al. (Greluk et al., 2016) studied the OSRE (H_2_O:EtOH:O_2_ = 9:1:0.7) using PtKCo/CeO_2_ catalyst, the temperature of 420°C was sufficient to achieve complete conversion but only maintained 5 h. Coke deposition that led to the removal of active sites from the catalyst surface was considered to be the major deactivation mechanism. The H_2_ distribution decreased from 68% to 60%, and the CO distribution increased from 2% to 2.5% with the increase of process time. Casanovas et al. (Casanovas et al., 2006) investigated the OSRE (H_2_O:EtOH:O_2_ = 13:1:0.5) using Pd/ZnO catalyst at low temperatures (300°C − 450°C), the ethanol could be converted completely. The H_2_ selectivity increased from 37% to 61%, and the CO selectivity decreased from 4.9% to 0.1% with increasing temperature. Palma et al. (Palma et al., 2017) surveyed the OSRE on mesoporous silica supported PteNi/CeO_2_ catalysts, which displayed stable behavior for 135 h under 500°C. Therefore, the fabricated Pt-Ru/ZrO_2_ showed potential as a candidate OSRE catalyst under low temperatures in this study.

## 4 Conclusion

This investigation indicated that the feed molar ratio and kinds of support have influence on the catalytic performance of PtRu-based catalysts. The optimal molar ratios of the H_2_O/EtOH and O_2_/EtOH feeds were 4.9 and 0.44, respectively for the OSRE reaction. In addition to the synergistic effect emerged between the metal oxide supports and PtRu active species, the reducible oxide supports also could provide the lattice oxygen to promote the oxidation of CO and the water gas shift reaction. Both the PtRu/ZrO_2_ and the PtRu/CeO_2_ catalysts appeared to be excellent OSRE catalysts to produce hydrogen under low temperatures with lesser distributions toward unwanted CO and CH_4_ with complete ethanol conversion. Evidently, the PtRu supported on ZrO_2_ and CeO_2_ exhibited superior catalytic performance in the OSRE reaction. It was quite stable during a long time evaluation of more than 80 h; the maximum Y_H2_ was maintained at 4.5–4.2 and the CO distribution approached 3.3–3.5 mol% around 84 h test at 340°C on the PtRu/ZrO_2_ catalyst.

## Data Availability

The original contributions presented in the study are included in the article/supplementary material, further inquiries can be directed to the corresponding authors.
